# Nutritional Assessment and Nutrient Supplement in Patients with Chronic Kidney Disease

**DOI:** 10.3390/nu15081964

**Published:** 2023-04-19

**Authors:** Masashi Mizuno

**Affiliations:** Department of Renal Replacement Therapy, Nagoya University Graduate School of Medicine, 65 Tsurumai-cho, Showa-ku, Nagoya 466-8550, Japan; mmizu@med.nagoya-u.ac.jp

Currently, aging is an important social problem globally. Renal function declines as a person ages, and chronic kidney diseases (CKD) caused by nephrosclerosis due to aging are increasingly considerable causes of end-stage renal disease (ESRD). In fact, in 2018, nephrosclerosis overtook chronic glomerulonephritis in Japan as the most prevalent causative disease requiring dialysis for ESRD [1].

Given this background, appropriate nutritional management will undoubtedly play a more important role in preventing the progression of renal function impairment than it has in the past. Nutritional management is not only a part of conservative therapies for CKD but also an essential aspect of care for CKD patients who have progressed to ESRD, even if on dialysis. Salt restriction, as well as appropriate protein, potassium, and/or phosphate intake should be the main considerations in nutritional management. However, protein restriction can be contentious considering the need to balance protecting residual renal function and avoiding sarcopenia in the present aging world. Another recent interesting point regarding the prognosis of patients with CKD has been the intake of rare metals, including the effects of nutrient supplements and associated agents. This Special Issue focuses on overall nutritional management from early stage CKD to stage 5 CKD requiring renal replacement therapy (i.e., ESRD).

This issue contains four interesting research articles [2,3,4,5] and two important review articles [6,7]. In our article, we present the usefulness of controlling nutritional status (COUNT) score for evaluating nutritional status in ESRD patients and show that nutritional status at the start of dialysis affects the prognosis of ESRD patients [2]. With the aging of societies, we may need to consider optimal nutritional management to prevent sarcopenia. Lin Y-L et al. propose an appropriate assessment of sarcopenia in ESRD patients on peritoneal dialysis (PD) [3]. Nutritional assessments to secure the required amount of nutrients under dietary restrictions, as well as proper management performance, are necessary. In ESRD patients on dialysis, particularly PD, uncontrolled body volume is indicative of poor prognosis. In their article, Ng ZJK-C et al. conclude that body composition assessments should be a part of comprehensive nutritional assessments [4]. In nutritional management, consideration must be given to the prevention of complications in CKD patients, particularly in patients with ESRD on long-term dialysis. Kuno T et al. focus on the progression of calcification of radial arteries in CKD patients because the progression of arterial calcification was found to correlate with a poor prognosis [5]. In their article, they show that low hemoglobin, low transferrin saturation, and high blood pressure are risk factors for arterial calcification, suggesting that nutritional management with iron supplements is not only important for controlling anemia but might also prevent arterial calcification.

In a review article [6], Kuma A et al. emphasize that nutritional management should attempt to control lifestyle-related risk factors to reduce the development and progression of CKD. Finally, Ashkar F et al. focus on the possible usefulness of polyphenols as additive ingredients to enhance mitochondrial function [7]. Both review articles point to the relationship between mitochondrial dysfunction and renal impairment [7,8], and this could be an important consideration because the use of polyphenols may be one means of mitigating mitochondrial dysfunction.

We believe this Special Issue will not only give readers valuable insight into nutritional assessment and nutrient supplementation in patients with CKD to support the daily medical care of these patients but also provide the impetus for future research.
